# Methodological evaluation of *Helicobacter pylori* infection and clarithromycin resistance detection via gastric fluid PCR

**DOI:** 10.3389/fcimb.2026.1744959

**Published:** 2026-02-10

**Authors:** Xiujuan Wang, Haocheng Wang, Jiahuan Gao, Zhenyu Zhang

**Affiliations:** Department of Gastroenterology, Nanjing First Hospital, Nanjing Medical University, Nanjing, Jiangsu, China

**Keywords:** clarithromycin, gastric juice, gastric mucosa, *Helicobacter pylori*, PCR

## Abstract

**Objective:**

To evaluate the clinical feasibility of using PCR testing based on gastric juice samples as an alternative to gastric mucosa samples for determining *Helicobacter pylori* (*H. pylori*) infection and clarithromycin resistance.

**Methods:**

A total of 470 patients who underwent upper gastrointestinal endoscopy and a rapid urease test (RUT) at our hospital between January 2025 and April 2025 were selected. Both gastric juice and gastric mucosa samples were collected from each patient. Fluorescent PCR melting curve analysis was used to detect *H. pylori* DNA and mutations at the 2142 or 2143 sites of the 23S rRNA gene in both sample types in order to assess *H. pylori* infection status and clarithromycin resistance.

**Results:**

Compared to RUT, PCR testing of gastric juice samples for determining H. pylori infection showed a sensitivity of 92.86% (95% CI: 89.6–95.3), specificity of 95.0% (95% CI: 89.5–97.9), positive predictive value (PPV) of 98.19% (95% CI: 96.1–99.3), negative predictive value (NPV) of 82.01% (95% CI: 74.6–87.8), and good agreement (Kappa = 0.835, P < 0.001). Using gastric mucosa PCR as the “gold standard,” PCR testing of gastric juice samples for H. pylori infection demonstrated a sensitivity of 93.90% (95% CI: 90.8–96.1), specificity of 93.65% (95% CI:87.9–97.0), PPV of 97.58% (95% CI: 95.2–98.9), NPV of 84.89% (95% CI: 77.7–90.3), and good agreement (Kappa = 0.848, P < 0.001). Using culture-based drug susceptibility testing as the “gold standard,” the resistance detection rate of gastric juice PCR was 86.42% (70/81), with moderate agreement (Kappa = 0.684, P < 0.001). The resistance detection rate of gastric mucosa PCR was 88.89% (72/81), with good agreement (Kappa = 0.788, P < 0.001). Using first-generation sequencing gene detection as the “gold standard,” the resistance detection rate of gastric juice PCR was 84.27% (75/89), with moderate agreement (Kappa = 0.694, P < 0.001). The resistance detection rate of gastric mucosa PCR was 87.64% (78/89), with good agreement (Kappa = 0.805, P < 0.001).

**Conclusion:**

Compared to gastric mucosa samples, PCR testing using gastric juice samples enables relatively non-invasive, rapid, efficient, and reliable diagnosis of *H. pylori* infection and clarithromycin resistance.

## 1 Introduction

*Helicobacter pylori* (*H. pylori*) is a common gastrointestinal bacterium closely associated with the pathogenesis of chronic gastritis, peptic ulcers, gastric mucosa-associated lymphoid tissue (MALT) lymphoma, and gastric cancer (Jiang et al., 2017). *H. pylori* infection is a communicable disease transmitted from person to person primarily via the oral-oral or fecal-oral route, with a global prevalence as high as 50% (Zhou et al., 2022). Current national and international consensus guidelines state that all infected patients should receive treatment unless contraindications exist (Malfertheiner et al., 2022; Helicobacter Pylori Study Group, 2022). The current consensus guidelines recommend bismuth-containing quadruple therapy (comprising one proton pump inhibitor [PPI], two antibiotics, and one bismuth agent) as the primary empirical regimen for eradicating *H. pylori*. In recent years, the eradication rate of *H. pylori* has declined, primarily due to increasing bacterial resistance to antimicrobial agents, particularly to clarithromycin, metronidazole, and levofloxacin (Kuo et al., 2017). To improve the *H. pylori* eradication rate, antimicrobial resistance testing can be performed prior to initiating eradication therapy. Traditional antimicrobial resistance testing relies on endoscopic biopsy of gastric mucosa for bacterial culture and drug susceptibility testing, which is characterized by stringent culture requirements, time-consuming procedures, and low culture success rates. In contrast, molecular biology and genetic testing techniques for detecting *H. pylori* infection and antimicrobial resistance offer rapid, timely results with high sensitivity and specificity (Pichon et al., 2020; Li et al., 2020). Previous studies have indicated good agreement between genotypic detection of clarithromycin resistance and phenotypic detection results (κ coefficient = 0.810) (Zhong et al., 2021; Zhang et al., 2020).

The polymerase chain reaction (PCR) is the most commonly used molecular biology detection method, known for its simplicity, rapidity, high sensitivity, and strong specificity. PCR can also detect *H. pylori* strains that are difficult to identify using conventional methods from samples other than gastric mucosa, such as gastric juice, oral cavity, and dental plaque. Simultaneously, it can assess *H. pylori* resistance, providing a basis for precise medication in patients infected with *H. pylori* (Wen et al., 2023). Gastric juice reflects the overall environment of the stomach, as *H. pylori* from different mucosal sites can enter it, making it unaffected by the patchy distribution of *H. pylori* in the stomach. Based on this rationale, this study employed the fluorescent PCR melting curve method to detect *H. pylori* infection and clarithromycin resistance genes in both gastric mucosa and gastric juice samples from patients. The aim is to evaluate the performance of molecular biology testing based on gastric juice samples, with the goal of guiding drug selection and facilitating precise treatment for patients.

## 2 Materials and methods

### 2.1 Study population

A total of 470 patients who required upper gastrointestinal endoscopy for various indications and underwent a rapid urease test (RUT) at our center between January 2025 and April 2025 were enrolled as study subjects. This cohort included both *H. pylori*-positive and *H. pylori*-negative individuals. Among the positive individuals, 131 patients consented to undergo *H. pylori* culture-based drug susceptibility testing and first-generation sequencing gene detection. This study was approved by the Medical Research Ethics Committee of our hospital (Approval No.: KY20250123-05), and written informed consent was obtained from all patients.

Inclusion Criteria: (1) Patients clinically diagnosed and initially assessed as having chronic gastritis and/or peptic ulcer; (2) Patients without contraindications for gastroscopy, such as gastrointestinal perforation, advanced age, or unstable vital signs; (3) Patients who agreed to undergo gastroscopy.

Exclusion Criteria included: (1) A history of prior *H. pylori* eradication therapy; (2) Use of antibiotics or bismuth agents within the past month, or acid suppressants within the past two weeks; (3) Diagnosis of gastric mucosa-associated lymphoid tissue (MALT) lymphoma or other malignant tumors; (4) A history of esophageal or gastric surgery; (5) Pregnancy or lactation; (6) Poor compliance or refusal to sign the informed consent form.

For all 470 patients, RUT and fluorescent PCR melting curve analysis were utilized to detect *H. pylori* infection and resistance genes. The sensitivity, specificity, positive predictive value, negative predictive value, and clarithromycin resistance rate of PCR testing based on gastric juice samples were calculated. The agreement between the test results from gastric juice samples and those from RUT and gastric mucosa testing was assessed using the Kappa test.

### 2.2 Sample collection and handling

#### 2.2.1 RUT detection

Under endoscopy, a disposable biopsy forceps was used to obtain one piece of gastric mucosa tissue (approximately 0.1 cm × 0.1 cm in size) from the gastric antrum, 2–3 cm from the pylorus. The tissue sample was placed into a rapid urease test diagnostic kit (Shanghai Huitai Medical Technology Co., Ltd.). Color changes were observed against a white background under natural light. A yellow color indicated a negative result, signifying no *H. pylori* infection; a light red or rose-red color indicated a positive result, confirming *H. pylori* infection. The results were assessed by an experienced clinician.

#### 2.2.2 Collection and processing of gastric fluid samples

Prior to the gastroscopic examination, approximately 2–3 mL (minimum 1 mL) of fasting gastric juice was aspirated through the endoscope’s biopsy channel using a disposable 20 mL syringe. The collected gastric juice was then transferred into a preservation tube for subsequent analysis via the fluorescent PCR melting curve method.

#### 2.2.3 Gastric mucosa collection and processing

Residual gastric mucosa samples from patients with RUT-confirmed *H. pylori* infection were collected and placed into preservation tubes. Subsequently, PCR amplification and hybridization with *H. pylori*-specific fluorescent probes were performed using a fully automated nucleic acid purification and fluorescence PCR analyzer. The instrument’s software system automatically monitored fluorescence intensity in real-time. By calculating real-time fluorescence variation, melting curves were generated, yielding fluorescence derivative values and melting temperatures for each sample, thereby enabling the detection and analysis of the tested samples.

#### 2.2.4 Fluorescent PCR melting curve method

The testing principle comprises two main components. The first part utilizes real-time fluorescent quantitative PCR technology, targeting the 23S rRNA gene region of *Helicobacter pylori*. Specific *H. pylori* amplification primers (HPC-F: 5’-GTGGAGGTGAAAATTCCTCCTACCC-3’, HPC-R: 5’-GGCTCCATAAGAGCCAAAGCCCTTAC-3’) are added, followed by denaturation and annealing steps. The KOD DNA polymerase, selected for its high-speed amplification capability, catalyzes the extension of each primer along the gene strand. The enzymatic activity, facilitated by dNTP substrates and magnesium ions, acts on the annealed primers to facilitate extension. This cycle is repeated to amplify the target gene strand. The amplified target gene strand hybridizes with a specifically matched *H. pylori* fluorescent probe (5’-CAAGACGGAAAGACCC-3’). The presence or absence of *H. pylori* is detected by monitoring the resulting fluorescence changes.

The second part leverages the difference in melting temperature (Tm) between *H. pylori* nucleic acid sequences with and without mutations at the 2142 and 2143 sites of the 23S rRNA gene. A melting curve is generated by detecting fluorescence changes, thereby determining whether a mutation is present at the 2142 or 2143 site of the *H. pylori* 23S rRNA gene. The interpretation criteria for the results are presented in **Table 1**.

**Table 1:** Interpretation criteria for fluorescent PCR melting curve results.

Result category		Fluorescence derivative value	Tm (°C)
*H. pylori*(+)	No Mutation	≥10	50-60
	Mutation	≥10	42-50
*H. pylori*(-)	Fluorescence Derivative Value < 10, and Internal Control Fluorescence Derivative Value ≥ 1.0		42-68

Tm refers to the melting temperature at the peak of the melting curve.

#### 2.2.5 *H. pylori* isolation, culture, identification, and drug susceptibility testing

The culture medium for *H. pylori* isolation consisted of Columbia agar supplemented with a selective agent mixture [vancomycin 250 mg/L, amphotericin B 200 mg/L, polymyxin 220 mg/L, trimethoprim (TMP) 300 mg/L] and 5% defibrinated sheep blood. The transport medium was composed of Brucella broth with 10% sucrose and 10% fetal calf serum. Three gastric mucosa biopsies (two from the lesser curvature of the gastric antrum and one from the gastric body) were obtained via endoscopy, placed into the transport medium, and immediately delivered to the laboratory. The samples were homogenized and inoculated onto *H. pylori* agar plates. A microaerophilic atmosphere (5% O_2_, 10% CO_2_, and 85% N_2_) was established using the gas-pack evacuation-replacement method, and the plates were incubated at 37°C for 72 hours under >95% humidity. Small, colorless, translucent colonies were selected for identification via Gram staining, urease test, and oxidase test.

Drug susceptibility testing was performed using the Kirby-Bauer (KB) disk diffusion method. The procedures and interpretations were conducted in accordance with the “Technical Standards for Antimicrobial Susceptibility Testing of Helicobacter pylori” (National Institute for Food and Drug Control [NIFDC], China, Version 2022). Clarithromycin resistance was defined as a zone diameter of ≤ 20 mm, which aligns with the breakpoint specified by the NIFDC and is consistent with the CLSI supplement M45 (3rd edition). The reference strain used was ATCC 43504 (obtained from Renji Hospital, Shanghai).

### 2.3 Statistical methods

Data and results were recorded in an Excel database. Statistical analysis was performed using SPSS software (version 23.0). The qualitative detection results of the two testing methods were summarized. The sensitivity, specificity, positive predictive value, and negative predictive value of the gastric juice PCR method were calculated. A Kappa test was conducted on the qualitative results to evaluate the agreement between the two methods. A Kappa value ≥ 0.75 indicated good agreement, a value between 0.4 and 0.7 indicated moderate agreement, and a value ≤ 0.4 indicated poor agreement.

## 3 Result

### 3.1 Detection rates and agreement of *H. pylori* in patient gastric mucosa and gastric juice

As shown in **Table 2**, among the 470 patients, 350 were RUT-positive and 120 were RUT-negative. Of the 350 RUT-positive patients, PCR testing of their gastric juice samples identified 325 as *H. pylori* DNA-positive and 25 as negative. Among the 120 RUT-negative patients, gastric juice PCR testing was positive in 6 cases and negative in 114 cases. Consequently, compared to RUT, PCR testing of gastric juice samples for determining H. pylori infection showed a sensitivity of 92.86% (325/350; 95% CI: 89.6%–95.3%), specificity of 95.0% (114/120;95% CI: 89.5%–97.9%), positive predictive value (PPV) of 98.19% (325/331;95% CI: 96.1%–99.3%), negative predictive value (NPV) of 82.01% (114/139; 95% CI: 74.6%–87.8%), and good agreement (Kappa = 0.835, P < 0.001).

**Table 2:** Diagnostic performance of gastric juice PCR for detecting *H. pylori*.

Diagnostic method (or reference test)		Gastric juice PCR		SEN(%) (95%CI)	SPE(%) (95%CI)	PPV(%) (95%CI)	NPV(%) (95%CI)	Overall concordance rate (%)	Kappa value	P value
		(+)	(-)							
RUT	(+)	325	25	92.86 (89.6-95.3)	95.0 (89.5-97.9)	98.19 (96.1-99.3)	82.01 (74.6-87.8)	93.40	0. 835	<0.001
	(-)	6	114							
Gastric Mucosa PCR	(+)	323	21	93.90	93.65	97.58	84.89	93.83	0.848	<0.001
	(-)	8	118	(90.8-96.1)	(87.9-97.0)	(95.2-98.9)	(77.7-90.3)			

NPV, negative predictive value; PPV, positive predictive value; SEN, sensitivity; SPE, specificity.

Among the 344 gastric mucosa PCR-positive samples, 323 tested positive and 21 tested negative by gastric juice PCR. Among the 126 gastric mucosa PCR-negative samples, 8 tested positive and 118 tested negative by gastric juice PCR. Therefore, using gastric mucosa PCR as the ‘gold standard,’ PCR testing of gastric juice samples for H. pylori infection demonstrated a sensitivity of 93.90% (323/344;95% CI: 90.8%–96.1%), specificity of 93.65% (118/126;95% CI: 87.9%–97.0%), PPV of 97.58% (323/331; 95% CI: 95.2%–98.9%), NPV of 84.89% (118/139; 95% CI: 77.7%–90.3%), and good agreement (Kappa = 0.848, P < 0.001).

### 3.2 *H. pylori* resistance detection rates and agreement in patient gastric mucosa and gastric juice

As shown in **Table 3**, among the included 470 patients, 131 underwent *H. pylori* culture and drug susceptibility testing. The culture-based drug susceptibility results indicated clarithromycin resistance in 81 patients, yielding a total resistance rate of 61.83% (81/131).

**Table 3:** Concordance analysis between phenotypic and genotypic antibiotic resistance testing.

Detection method/Concordance measure	Test result	Antibiotic resistance testing					
		Culture			First-generation sequencing		
		Susceptible	Resistant	Negative	Susceptible	Resistant	Negative
Gastric fluid PCR	Susceptible	29	5	0	29	11	1
	Resistant	11	70	0	5	75	0
	Negative	1	5	10	0	3	7
Kappa		0.684			0.694		
*P* value		<0.001			<0.001		
Susceptible	32	8	0	32	8	0
Gastric mucosa PCR	Resistant	0	73	5	0	78	0
	Negative	2	0	11	2	3	8
	Kappa		0.788			0.805		
*P* value		<0.001			<0.001		

Using the culture-based susceptibility results as the “gold standard,” the PCR detection of 23S rRNA gene mutations was compared. Gastric juice PCR identified resistance in 70 cases, with a resistance detection rate of 86.42% (70/81). The agreement between the two methods was moderate (Kappa = 0.684, P < 0.001). Gastric mucosa PCR identified resistance in 72 cases, with a resistance detection rate of 88.89% (72/81), demonstrating good agreement with the gold standard (Kappa = 0.788, P < 0.001).

Using first-generation sequencing results as the “gold standard,” gastric juice PCR identified resistance in 75 cases, with a resistance detection rate of 84.27% (75/89). The agreement was moderate (Kappa = 0.694, P < 0.001). Gastric mucosa PCR identified resistance in 78 cases, with a resistance detection rate of 87.64% (78/89), demonstrating good agreement (Kappa = 0.805, P < 0.001).

## 4 Discussion

The Fifth National Consensus Report on the Management of *Helicobacter pylori* Infection recommends seven different bismuth-containing quadruple therapy regimens, combining various antibiotics, as the primary empirical treatment for eradicating *H. pylori*. The included antibiotics are clarithromycin, metronidazole, levofloxacin, amoxicillin, furazolidone, and tetracycline. However, in clinical practice, the high resistance rate to metronidazole, poor clinical accessibility of tetracycline, and the relatively high risk of adverse reactions associated with furazolidone limit their utility. Consequently, clarithromycin is widely recommended for the first-line treatment of *H. pylori* infection. In recent years, with the extensive use of antibiotics, resistance of *H. pylori* to antibiotics has been increasing annually, particularly to clarithromycin. In China, the primary resistance rate of *H. pylori* to clarithromycin ranges from 20% to 50% (Hu et al., 2017). The Maastricht VI/Florence Consensus recommends that routine drug susceptibility testing (either by bacterial culture or molecular biology methods) for antibiotics like clarithromycin should be performed prior to the initial treatment of *H. pylori* infection to guide therapy (Malfertheiner et al., 2022).

Current research indicates that the mechanism of clarithromycin resistance in *H. pylori* involves point mutations in the peptidyl transferase region (Domain V) of its 23S rRNA gene. These mutations alter the ribosomal conformation, thereby inhibiting the binding of clarithromycin to *H. pylori*. As a result, the drug fails to prevent bacterial protein synthesis, leading to the development of resistance. The primary point mutations include A2142C, A2142G, A2143G, A2143C, and T2182C, among which the first three are the most prevalent mutation sites (Tai et al., 2022; Liu et al., 2015). Consequently, detecting mutations at these relevant gene loci via PCR can determine clarithromycin resistance.

Previous non-invasive molecular biology testing has primarily focused on stool samples. While stool samples are relatively easy to collect, the extraction of *H. pylori* DNA from them is influenced by several factors. On one hand, the nucleic acid content of *H. pylori* in stool is extremely low and prone to degradation. On the other hand, the abundance of intestinal microorganisms can interfere with the nucleic acid amplification process. Furthermore, impurities present in stool samples can significantly impair the efficiency of real-time PCR amplification (Ren et al., 2025; İnal et al., 2025). In particular, the findings reported by İnal et al. (2025) demonstrate that inhibitory substances in stool samples may adversely affect amplification efficiency and diagnostic performance when using commercial PCR kits.

In recent years, several studies have explored PCR-based detection of *H. pylori* using gastric juice samples (Soh et al., 2023; Wang et al., 2024; Tsuda et al., 2022). Gastric juice represents the overall gastric environment and is unaffected by the patchy distribution of *H. pylori* within the stomach. Moreover, compared to gastric mucosa biopsy, gastric juice PCR testing is less invasive. This is particularly advantageous for patients on anticoagulant therapy (e.g., aspirin, clopidogrel) who are unsuitable candidates for mucosal biopsy. Beyond detecting the presence of *H. pylori* infection, gastric juice PCR can also determine clarithromycin resistance (Han et al., 2023; Kakiuchi et al., 2022), enabling personalized treatment based on the resistance profile (Weng et al., 2023) Additionally, compared to culture and drug susceptibility testing performed on gastric mucosa biopsies, gastric juice PCR is more cost-effective and has a shorter turnaround time (Kuo et al., 2016).

This study employed the fluorescent PCR melting curve method to detect *H. pylori* infection and clarithromycin resistance genes in both gastric mucosa and gastric juice samples from patients. This method utilizes KOD DNA polymerase for *H. pylori* detection. Compared to Taq DNA polymerase, which is commonly used in standard PCR systems, KOD DNA polymerase exhibits greater thermostability, a faster synthesis rate, and a lower error rate. The use of KOD DNA polymerase enables the entire PCR detection process to be completed within 1 hour, significantly improving detection efficiency compared to the 4 to 6 hours typically required by conventional PCR methods (Huang et al., 2019).

The rapid urease test (RUT) is simple, fast, and relatively accurate. It allows for *H. pylori* detection results to be obtained within a short time frame during gastroscopy. Consequently, the Maastricht V consensus recommends RUT as a first-line diagnostic method for *H. pylori* infection in patients with an indication for endoscopy and no contraindications for biopsy, and it is widely used in clinical practice (Uotani and Graham, 2015; Malfertheiner et al., 2022).

This study evaluated the feasibility of PCR testing based on gastric juice samples for detecting *H. pylori* infection by comparing the results with those of the RUT. The results demonstrated that gastric juice PCR testing achieved high sensitivity and specificity. Compared to RUT, the overall concordance rate was 93.40%, indicating good agreement between the two methods (Kappa = 0.835, P < 0.001). When compared to gastric mucosa PCR, the overall concordance rate was 93.83%, also indicating good agreement (Kappa = 0.848, P < 0.001). A previous study reported a 95.9% agreement between *H. pylori* detection results from gastric juice samples and the ¹³C-urea breath test (¹³C-UBT) (Han et al., 2023). These findings indicate that PCR testing based on gastric juice samples yields satisfactory performance compared to both the most commonly used invasive and non-invasive clinical methods.

In evaluating clarithromycin resistance using gastric juice samples, comparisons were made not only with the clinical “gold standard” of culture-based drug susceptibility testing but also with first-generation sequencing results. The results showed that, compared to the culture-based susceptibility results, gastric juice PCR achieved a clarithromycin resistance detection rate of 86.42%, with good agreement between the two methods (Kappa = 0.788, P < 0.001). Compared to the first-generation sequencing results, gastric juice PCR had a resistance detection rate of 84.27%, demonstrating moderate agreement (Kappa = 0.694, P < 0.001). These findings indicate that in this study, the genotypic detection of clarithromycin resistance by gastric juice PCR showed good agreement with phenotypic resistance detection results, which is largely consistent with previous studies (Zhong et al., 2021; Zhang et al., 2020). Furthermore, genotypic resistance testing via gastric juice PCR was also feasible in individuals with *H. pylori* infection who tested negative by culture.

This study benefited from a relatively large sample size and the simultaneous analysis of both gastric juice and gastric mucosa specimens from the same patients. This approach minimized the confounding effects arising from different sample sources and enhanced the reliability of our findings. However, this study has certain limitations. As a single-center investigation, the generalizability of the results may be limited. Future research is needed to validate these findings across different regions and populations. Secondly, this study only assessed genotypic resistance to clarithromycin and did not evaluate resistance to other antibiotics. This was primarily due to the current inconsistencies between genotypic and phenotypic resistance for other antibiotics used in *H. pylori* eradication. These inconsistencies stem from factors such as the scattered distribution and low representation of resistance gene mutation sites, as well as non-genetic mechanisms of resistance (Tshibangu-Kabamba and Yamaoka, 2021). Future studies could focus on developing detection methods for a broader range of resistance gene loci to provide more options for personalized treatment. Furthermore, this study used RUT results as the standard for diagnosing *H. pylori* infection. However, the accuracy of RUT is influenced by factors such as the bacterial density in the biopsy specimen, detection time, and temperature. False-negative results may occur if the bacterial count in the biopsy specimen is below 1×10^4^, and since *H. pylori* exhibits a patchy distribution within the stomach, multiple biopsies can improve detection accuracy (Hsu et al., 2010).

In summary, this study employed the fluorescent PCR melting curve method to evaluate the detection of *H. pylori* infection and clarithromycin resistance using gastric juice samples, with concurrent comparison to results from gastric mucosa samples of the same individuals. The findings demonstrate that both gastric mucosa and gastric juice samples exhibited excellent detection performance. Furthermore, compared to gastric mucosa samples, PCR testing utilizing gastric juice samples enables a relatively non-invasive, rapid, efficient, and reliable diagnosis of *H. pylori* infection and clarithromycin resistance.

## Data Availability

The data that support the findings of this study are available on request from the corresponding author. The data are not publicly available due to privacy or ethical restrictions.
